# Breastfeeding success with the use of the inverted syringe technique for management of inverted nipples in lactating women: a study protocol for a randomized controlled trial

**DOI:** 10.1186/s13063-019-3880-8

**Published:** 2019-12-16

**Authors:** Mona Nabulsi, Rayan Ghanem, Marlie Abou-Jaoude, Ali Khalil

**Affiliations:** 1 Department of Pediatrics and Adolescent Medicine, Faculty of Medicine, American University of Beirut, Beirut, Lebanon; 2 Department of Obstetrics and Gynecology, Faculty of Medicine, American University of Beirut, Beirut, Lebanon

**Keywords:** Inverted nipple, Breastfeeding, Inverted syringe technique

## Abstract

**Background:**

Breastfeeding provides ideal infant nutrition, conferring several health benefits to children and their mothers. Women with inverted nipples, however, face difficulties that force them to prematurely terminate breastfeeding. Whereas available conservative measures for the correction of inverted nipples are of limited success, the use of an inverted syringe may be effective in achieving high rates of infant latching and exclusive breastfeeding. This technique, however, has not been investigated in a clinical trial.

**Methods/design:**

This open-label randomized controlled trial aims to investigate whether, in women with inverted nipples, the use of an inverted syringe increases the rate of exclusive breastfeeding at one month compared to standard care. One-hundred healthy women with grade 1 or 2 inverted nipples will be recruited as of 37 weeks of gestation. They will be randomly allocated to standard care (control group) or to an intervention group. The intervention consists of using an inverted syringe to evert the nipple before every breastfeed, starting with the first feed after delivery. The primary outcome measure is the rate of exclusive breastfeeding at 1 month. Secondary outcome measures include exclusive breastfeeding rates at 3 and 6 months, nipple eversion rate, successful latching rate, rates of any breastfeeding at 1, 3, and 6 months, breastfeeding-associated complications, maternal satisfaction with breastfeeding, maternal quality of life, and adverse events. Descriptive and regression analysis will be conducted under the intention to treat basis.

**Discussion:**

The use of the inverted syringe to evert inverted nipples is a simple, inexpensive, and safe technique that can be performed by mothers with inverted nipples. Findings of this trial, if positive, will provide much needed evidence for a safe, affordable, readily available, and simple intervention to treat inverted nipples, and improve breastfeeding practice among affected women.

**Trial registration:**

ClinicalTrials.gov, NCT03529630. Registered May 8, 2018.

## Background

Breastfeeding confers several health benefits to infants and their mothers, including protection from infectious and chronic diseases, improved child cognition, development, and intelligence, and decreased risks of maternal depression and malignancies [1–5]. As such, breastfeeding is recommended as the exclusive nutrition for the first 6 months of the infant’s life, with continuation until 2 years complemented with solid foods [6–8]. However, women with inverted nipples often have difficulties in maintaining breastfeeding due to improper infant latching that my cause insufficient milk extraction and poor infant satiety, thus leading to early termination of breastfeeding. Congenital inversion of the nipple, the most common nipple deformity, is due to early developmental arrest [9], with an estimated prevalence of about 10% [10]. However, nipple inversion can also be acquired secondary to mastitis, macromastia, carcinoma, or breast reduction, or can be associated with congenital syndromes such as Robinow and MR/MCA syndromes [11]. Han and Hong categorized the severity of inverted nipple into three grades. In grade 1, the nipple is easily pulled out manually, maintains good projection, and has minimal fibrosis. Grade 2 includes the majority of inverted nipple cases in which the affected nipple can be pulled out manually but fails to maintain projection and has moderate fibrosis beneath it. Grade 3 constitutes the rarest type of inverted nipples, which cannot be pulled out manually due to severe fibrosis [12].

The main treatment of inverted nipples is corrective surgery with sectioning of the lactiferous ducts. However, surgery is reserved for the severe grades such as invaginated nipples that are difficult to extract manually (grade 3), and it is not indicated for mild umbilicated nipples that can be momentarily extracted from their inverted positions (grades 1 and 2) [12]. Several non-surgical interventions have been used to extract inverted nipples. Hoffman exercises and the use of Woolwich breast shields, which were routinely recommended, have been shown to be of limited success [13]. The Niplette™ (Philips Avent, Andover, MA, USA) is another simple device that uses gentle negative suction to stretch the lactiferous ducts and extract the nipple with a success rate close to 80% [14]. It consists of a nipple cover, valve, and syringe and is operator dependent with one Niplette needed for each breast [14]. A more recent intervention was described by Chakrabarti and Basu in which a rubber band was put around the nipple base using a syringe applicator with a success rate of 62% [15]. However, this method carries with it certain risks, such as nipple injury, infection, or slipping of the band into the infant’s mouth [15]. The simplest, least expensive, most readily available, and most effective method to extract nipples has been reported in a case series by Kesaree et al. [16]. In this method, women with inverted nipples are trained to use an inverted syringe and apply gentle negative pressure around the nipple to evert it. The procedure can be repeated before each breastfeed as long as required. Of the eight women reported by Kesaree et al., seven (87.5%) infants succeeded in latching within 2 to 7 days, and six (75%) were exclusively breastfeeding at 6 weeks [16]. The main advantage of the inverted syringe over the Nipplette™ and the rubber and syringe technique is the fact that syringes are readily available in all countries, inexpensive, safe, and simple to use. To our knowledge, breastfeeding success with the use of the inverted syringe has not been previously investigated in a clinical trial. In this study, we aim to conduct the first randomized controlled trial to investigate whether the use of the inverted syringe in healthy term pregnant women with inverted nipples improves breastfeeding initiation and continuation rates, as compared to standard care.

## Methods/design

### Study design

The study protocol is designed in accordance with the Standard Protocol Items: Recommendations for Interventional Trials (SPIRIT; Additional file 1) [17]. This is a single-center, open-label, parallel-arm, randomized clinical trial that aims at investigating whether nipple suction exercises using the inverted syringe technique will improve exclusive breastfeeding rates at 1 month postpartum in women with inverted nipples.

### Study population

Eligible subjects are healthy pregnant women, at or above 18 years of age, with grades 1 or 2 inverted nipples. Inclusion criteria are gestation at 37 weeks or more, intention to breastfeed, and residing in Lebanon for 6 months after delivery. We will exclude women with grade 3 inverted nipples, previous breast surgery affecting the breast anatomy, high risk pregnancies, medical conditions that may interfere with breastfeeding, including a critical maternal condition, newborns with congenital malformations such as esophageal atresia, cleft lip, and/or palate, and women choosing artificial milk as their preferred infant nutrition. Women with term twin gestation will not be excluded.

### Recruiting process

Eligible pregnant women will be recruited from the Women’s Health Center and the Delivery Suite of the American University of Beirut Medical Center, Beirut, Lebanon. An inverted nipple is defined as a condition in which the nipple is pulled inward into the breast instead of pointing outward, classified according to Han and Hong [12]. During regular working hours, a trained research assistant will recruit eligible women after explaining the trial’s objective and procedures and verifying inclusion criteria. Written informed consent will be obtained from all participants.

### Randomization

We will randomly allocate eligible women to one of two parallel groups (experimental and control) in a 1:1 ratio according to a computer-generated random sequence. An independent statistician will prepare a set of sequentially numbered opaque sealed envelopes of the allocation group according to the generated random sequence to preserve randomization concealment. A participant’s group allocation will be revealed after verifying that the inclusion/exclusion criteria are met, and after written consent is obtained on the first day postpartum. This will avoid any selection bias introduced by the investigator knowing the allocation of the next subject.

### Description of the interventions

#### Control group

Women in the control group will receive standard medical care during pregnancy and postpartum as dictated by their obstetricians. Any advice regarding infant nutrition or treatment of inverted nipples will be left to the primary physician, including possible use of the syringe technique, in which case the participant will continue in the study and will be analyzed in her group as dictated by the intention to treat principle. We will provide the participants in the control group with documentation on breastfeeding-associated complications, use of artificial devices to correct the inverted nipple, such as nipple shields, Niplette, inverted syringe, or breast pump, and the number of artificial milk feeds given to the newborn with their justification in a diary. The research assistant will send weekly reminders to the participants in the control group using e-mail, telephone messages, or communication through social media, to remind them about documentation in the diary.

#### Intervention group

We will train the participants allocated to the experimental group on the use of the inverted syringe before each breastfeed as of the first postpartum day. We will also provide them with documentation on the daily use of the syringe, adverse events, breastfeeding-associated complications, use of artificial devices to correct the inverted nipple, the number of artificial milk feeds given to the newborn with their justification in a diary. The research assistant will send weekly reminders to the participants in the experimental group to use the syringe technique before each breastfeed using e-mail, telephone messages, or communication through social media. Syringe use will continue for as long as needed by the mother.

### Outcome measures

#### Main outcome measure

The trial’s primary outcome is the difference between the two groups in the rates of exclusive breastfeeding at 1 month postpartum, whether breast milk is provided to the infant through direct latching on the mother’s breast or as expressed milk offered by cup or bottle.

#### Secondary outcome measures

**Fig. 1:**
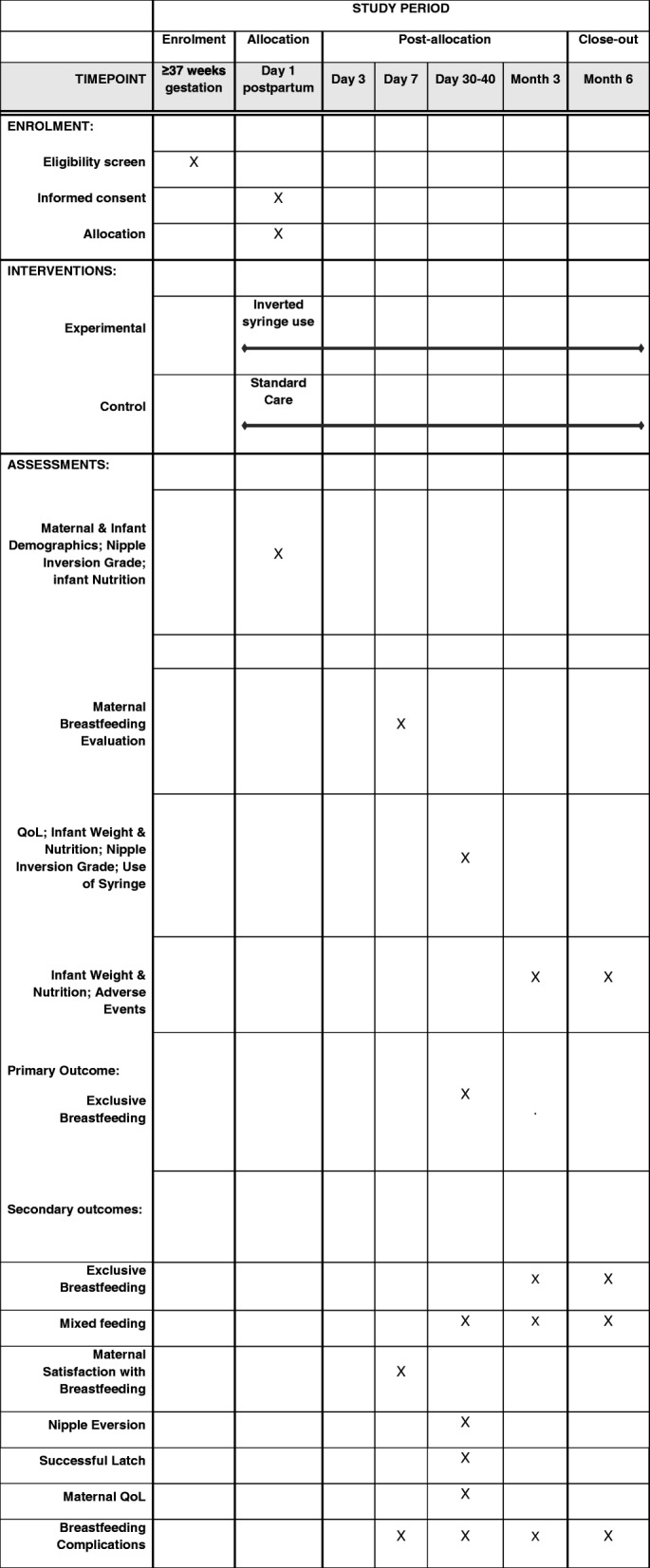
Schedule of enrolment, interventions, and assessments

Secondary outcomes include differences between the two groups in the rates of nipple eversion at 1 month, successful latching of the infant on mother’s breast at 1 month, exclusive breastfeeding at 3 and 6 months, mixed feeding at 1, 3, and 6 months, breastfeeding-associated complications such as sore nipple, mastitis, pain, bleeding, and breast engorgement at 1 week, 1, 3, and 6 months postpartum. Other secondary outcomes are maternal satisfaction with breastfeeding assessed at 1 week postpartum using the validated Maternal Breastfeeding Evaluation Scale [18, 19], and maternal quality of life assessed at 1 month using the validated Postpartum Quality of Life instrument [20] (Fig. 1).

### Data collection

At baseline, we will collect information on socio-demographics such as maternal age, parity, education, employment, family monthly income, previous breastfeeding, and longest duration of previous breastfeeding if present. Data on gestational age, mode of delivery, newborn’s gender, birth weight, APGAR score, and nursery admission status (regular vs intensive care), as well as grading of maternal nipple inversion will be recorded.

On the first day postpartum, the following information will be collected: exclusive breastfeeding (yes/no), use of artificial milk (number of feeds in 24 h, justification), sore nipple (yes/no), pain while breastfeeding (yes/no), compliance with the use of the syringe technique before each breastfeed (experimental group only), and the use of other conservative methods to pull out the nipple (in both groups).

On the third and seventh days postpartum, we will call the participants to remind them about documentation in the diary, collect data about maternal breastfeeding evaluation (day 7 only), and address any questions.

The diary will be collected at 30 and 40 days during the participant’s postpartum visit to her obstetrician. We will also administer the Postpartum Quality of Life instrument, and collect data on the infant’s weight at month 1 from the infant’s health record. The eversion of the nipple will also be assessed by the research assistant during this visit.

On months 3 and 6 postpartum, we will call the participants to collect information on infant feeding methods, adverse events, and infants’ weight at 3 and 6 months.

### Data management and quality assurance

The research assistant will be trained on how to approach eligible women, and how and when to contact them for follow up and data collection. Training entails familiarizing her with all the necessary documentation, including enrollment and consent forms, and consenting in accordance with the ethical principles of the Belmont Report. Demonstration and training on the use of the syringe will also be performed.

### Sample size

Based on data from a previous breastfeeding clinical trial that we conducted, 45% of women continue to breastfeed exclusively for 1 month postpartum [21]. We hypothesize that in women with inverted nipples, 40% of the experimental group and 5% of the control group will be exclusively breastfeeding at 1 month. To have 90% power and 5% type I error, and using the online calculator available at http://powerandsamplesize.com/Calculators/Compare-2-Proportions/2-Sample-Equality, we will need 25 women in each group to detect this 35% difference in the exclusive breastfeeding rates between the two groups.

In our recent breastfeeding support trial [21], the attrition rate was 40%. Since women with inverted nipples face more difficulties in breastfeeding than women with normally everted nipples, we hypothesize that 50% attrition rate would be a reasonable assumption for the proposed study. To account for a potential attrition rate of 50%, we inflated the sample size to become 100 women in total.

### Statistical methods

We will compare continuous variables using Student’s *t* test and categorical variables using Chi square test. Non-parametric tests will be used to analyze variables with skewed distribution or when the number of analyzed subjects is less than 30. We will calculate the crude rates of exclusive breastfeeding and mixed feeding at 1, 3, and 6 months in both groups. The 95% confidence intervals for the crude rates of breastfeeding will be computed using the normal approximation to the binomial distribution. We will also conduct multivariable logistic regression analysis to investigate the association between exclusive breastfeeding at 1 month (dependent variable) and the use of the inverted syringe (predictor) adjusting for potential confounders such as the grade of inverted nipple. Missing data will be imputed using last observation carried forward for the breastfeeding outcomes at 1, 3, and 6 months if breastfeeding was discontinued on last follow-up, the average value for continuous variables, and random replacement to maintain proportions for categorical variables, as appropriate. We will conduct sensitivity analysis with and without the imputed missing data to assess the impact of missing data on the primary outcome. We will also conduct repeated measurement analyses using generalized estimating equations (GEE) to explore the effect of the intervention over time. Blinded, intention to treat analysis will be conducted using the Statistical Package for Social Sciences (SPSS) version 24. A *p* value of < 0.05 will indicate statistical significance.

### Ethics approval

This study is approved by the Institutional Review Board (IRB) of the American University of Beirut. Written informed consent will be obtained from all participants. Since the study involves using the inverted syringe technique and documentation of relevant data in a diary, we estimate that the risks to women from participating in this study are negligible, not exceeding those of current standard practice. The only risk from nipple manipulation in the experimental group is the rare possibility of uterine contractions and labor induction if done prior to delivery. Since participants will start using the syringe right after delivery, this risk is eliminated. We also anticipate that the use of the syringe will result in less pain and less sore or bleeding nipples in the experimental group compared to the control group, who are at much greater risk for these complications secondary to poor infant latch. We will collect information on any adverse events during follow-up of participants, and will report all adverse events to the IRB as per institutional policies. Should any adverse events result directly from this study, the investigators’ institution will cover the cost of treating, on its premises, those medical adverse events.

Confidentiality of participants will be secured by storing the hard copies of the data collection forms in locked cabinets in the principal investigator’s office (MN). Access to electronic data stored in the SPSS data file will be password-protected, and restricted to the principal investigator (MN). The blinded analyst will be provided with a de-identified dataset to preserve confidentiality. Data will be stored for 5 years after publication of the study findings.

We will report any important modifications of the protocol to the IRB, trial participants, and trial registry. The trial’s results will be disseminated to healthcare professionals and the public through publication in a refereed journal, trial registry, and medical conferences.

## Discussion

This trial aims to investigate breastfeeding success with the use of the inverted syringe technique in women with grade 1 or 2 inverted nipples. We anticipate that this technique will increase the rates of direct infant latch on mother’s breast and exclusive and any breastfeeding continuation, will reduce maternal breastfeeding-associated complications such as sore or bleeding nipples, breast engorgement, or mastitis, and will improve maternal satisfaction with breastfeeding and maternal quality of life.

The main strength of our study is its design, as it will be the first trial to investigate the effectiveness of the inverted syringe in breastfeeding women with inverted nipples. However, it may be limited by its open-label status, which may increase the risks of performance and detection biases. Given the nature of the intervention, it would be difficult to blind the participants. Although the sample size is only 100 women, the study is powered to detect the hypothesized 35% difference in 1-month exclusive breastfeeding rates between the two groups, taking into account a potential high attrition rate. Another limitation is the fact that the study is conducted in a single center, which may impact its generalizability. However, women with inverted nipples face similar problems, irrespective of the context. Also, the intervention is readily available to women everywhere and is affordable. If the findings of this trial are positive, then replication of the study will still be needed in other contexts.

### Trial status

This is the first version of the study protocol dated May 8, 2018, the day it received approval from the IRB. Participant recruitment began on June 7, 2018 and is expected to be completed in 2 years.

## Supplementary information

**Additional file 1.** SPIRIT 2013 Checklist: Recommended items to address in a clinical trial protocol and related documents*.

## Data Availability

The de-identified dataset will be stored in a public data repository for future data sharing.

## Abbreviations

MR/MCA: Mental retardation/multiple congenital anomalies
SPSS: Statistical Package for Social Sciences
